# Supplementary material for: Optimization of robotic liquid handling as a capacitated vehicle routing problem
Source: Digit Discov. 2025 Aug 4;4(9):2593–601. doi: 10.1039/d5dd00233h (PMC12360158; doi:10.1039/d5dd00233h)
Supplement: DD-004-D5DD00233H-s008 [file DD-004-D5DD00233H-s008.pdf]

Supplementary Information (SI) for Digital Discovery.
This journal is © The Royal Society of Chemistry 2025

Supporting Information for Optimization of Robotic Liquid Handling as a Capacitated Vehicle Routing Problem

Guangqi Wu, Runzhong Wang, Connor W. Coley*

Contents

1 Simulation and Operation Parameters **2**

2 Additional Figures **3**

1 Simulation and Operation Parameters

All simulations were conducted using EvoSim. Key operational parameters are summarized in Table S1.

Table S1: Key liquid handling simulation parameters in EvoSim.

Parameter	Value
Aspiration speed	100 μL/s
Aspiration delay	500 ms
Dispensing speed	100 μL/s
Dispensing delay	500 ms
Tip retraction speed	20 mm/s

Table S2: Key liquid handling parameters of JANUS liquid handling workstation.

Parameter	Value
Aspiration speed	As specified
Aspiration delay	500 ms
Dispensing speed	As specified
Dispensing delay	500 ms
Scan in speed	150 mm/s
Scan out speed	150 mm/s
Retract from liquid speed	100 mm/s
Retract from liquid hight	10 mm

2 Additional Figures

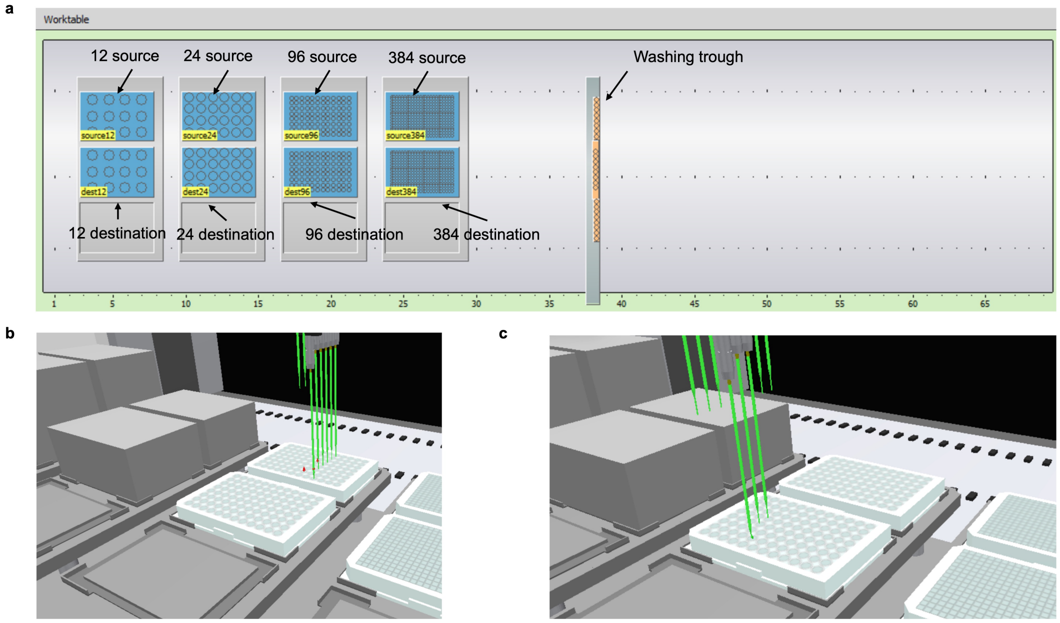

Figure S1: (a)Worktable layout for simulation. (b) Snapshot of aspirating from 96-well plate. (c) Snapshot of depensing to 96-well plate.

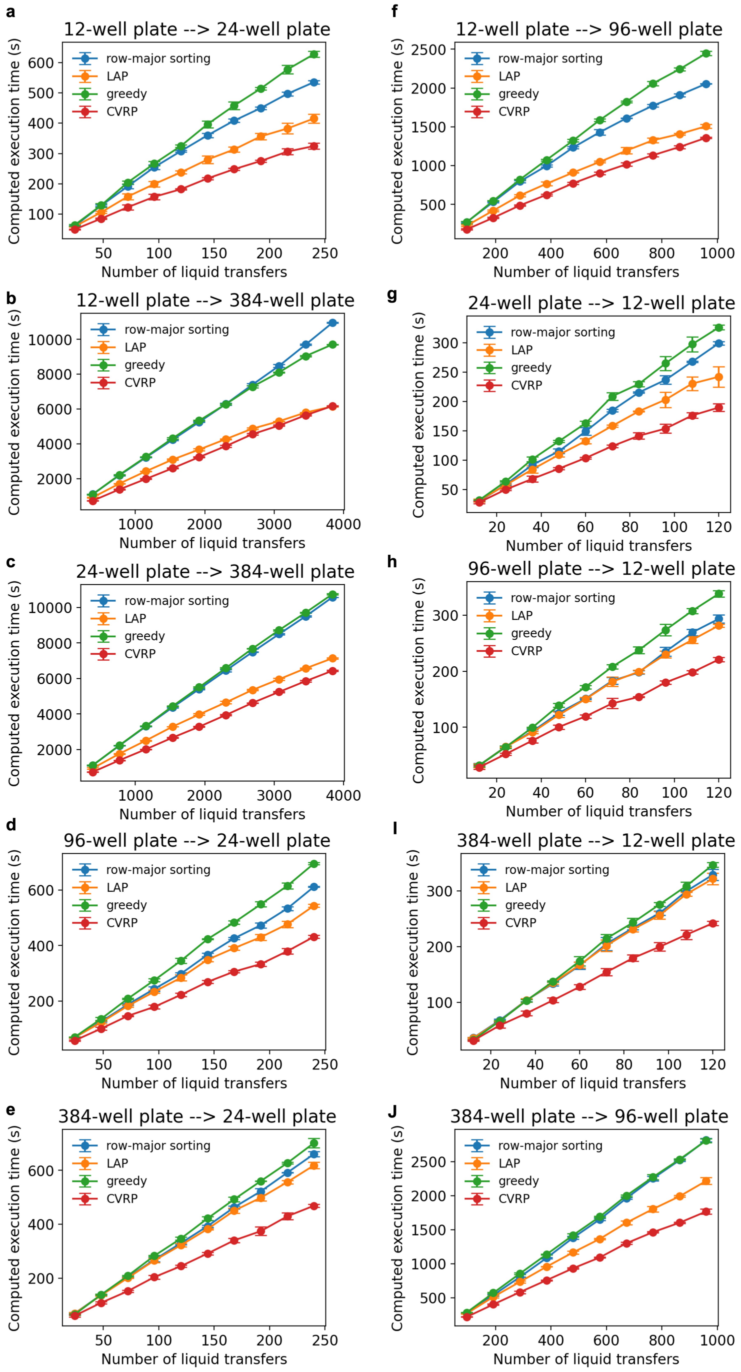

Figure S2: Execution time across different labware formats for random tasks.(a) from 12-well plate to 24-well plate, (b) from 12-well plate to 384-well plate, (c) from 24-well plate to 384-well plate, (d) from 96-well plate to 24-well plate, (e) from 384-well plate to 24-well plate, (f) from 12-well plate to 96-well plate, (g) from 24-well plate to 12-well plate, (h) from 96-well plate to 12-well plate, (i) from 384-well plate to 12-well plate, and (j) from 384-well plate to 96-well plate. For a given number of liquid transfers and labwares, 3 random tasks were generated and scheduled using different methods. The solving time for CVRP is 20 seconds. For each labware combination, we evaluated 10 different task sizes, with the number of liquid transfers set to multiples (from 1× to 10×) of the number of wells in the destination plate.

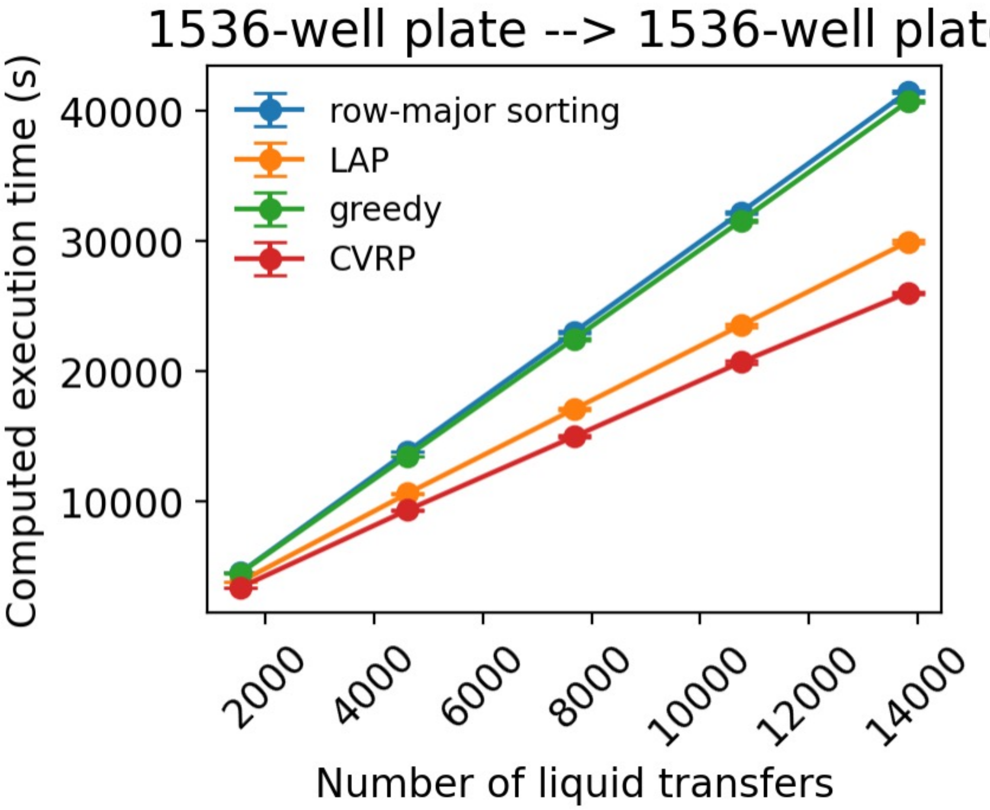

Figure S3: Performance of different methods on 1536-well plates. For a given number of liquid transfers, 3 random tasks were generated and scheduled using different methods. The solving time for CVRP is 60 seconds for tasks with less than 7000 transfers and 120 seconds for tasks with more than 7000 transfers. We evaluated 5 different task sizes (1536, 4608, 7680, 10752 and 13824).

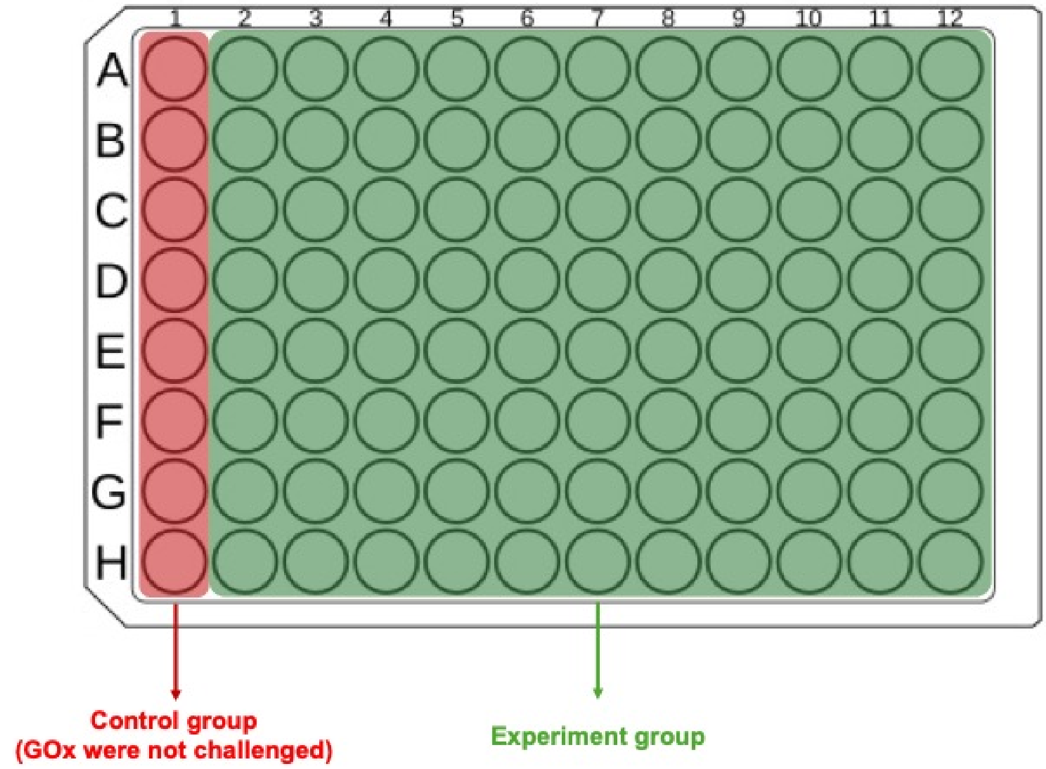

Figure S4: The layout of the 96-well plate during the autonomous optimization of polymer blends for enzyme stability.

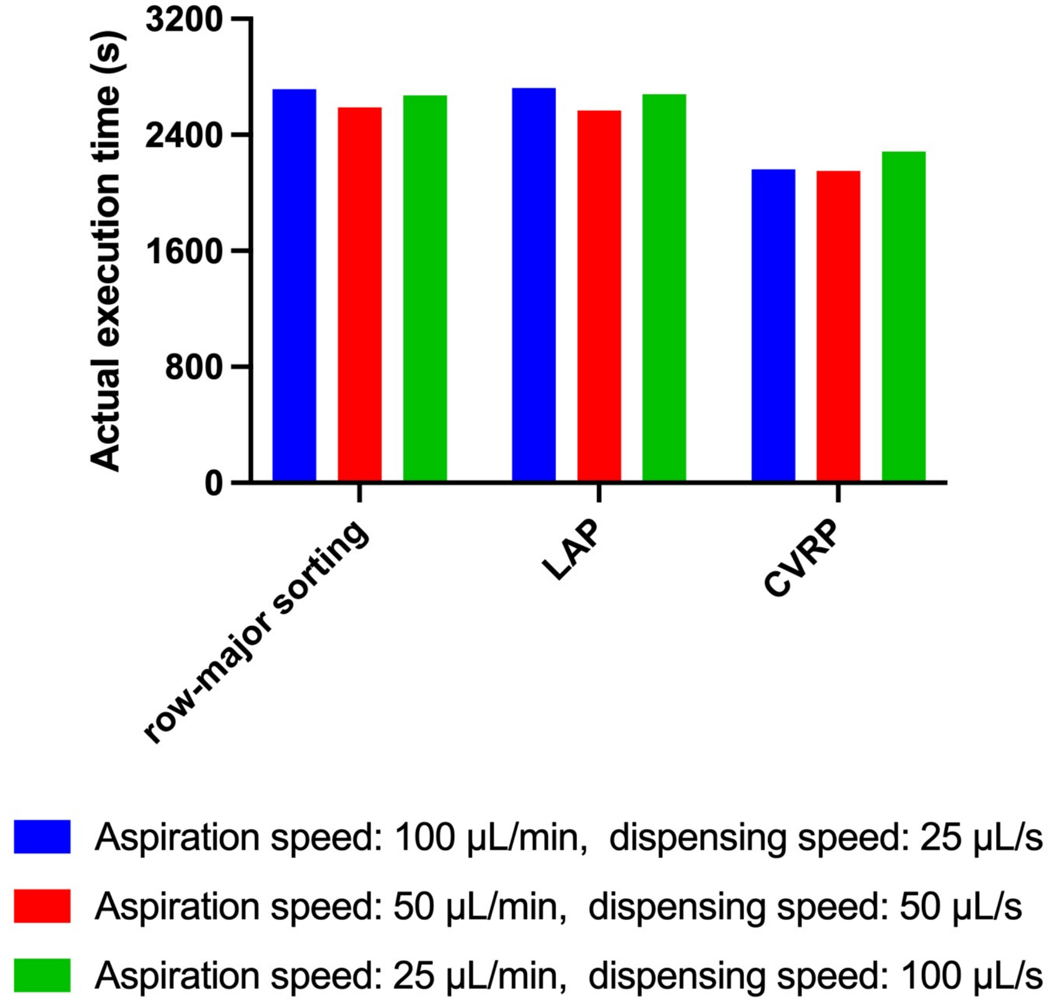

Figure S5: The actual execution time on JANUS liquid handling workstation of iteration 3 in Figure 7 scheduled with different methods. The optimization effect was tested according to the parameters specified in Table S2, with the specified aspiration and dispensing speeds. The CVRP-based method demonstrated superior performance compared to all other methods in various speed combinations.
